# A qualitative study of how structural vulnerability shaped COVID-19 testing behaviors in Portland, Maine

**DOI:** 10.3389/fpubh.2024.1433476

**Published:** 2024-10-17

**Authors:** Michael R. Kohut, Gloria D. Sclar, Leslie Nicoll, Caroline Fernandes, Andrew Volkers, Ann Tucker, Elizabeth A. Jacobs, Kathleen M. Fairfield

**Affiliations:** ^1^MaineHealth Institute for Research, Scarborough, ME, United States; ^2^Department of Public Health and Community Medicine, Tufts University, Boston, MA, United States; ^3^Friends of the Portland Community Free Clinic, Portland, ME, United States; ^4^Preble Street, Portland, ME, United States; ^5^Greater Portland Health, South Portland, ME, United States; ^6^Dell Medical School, University of Texas, Austin, TX, United States; ^7^Department of Medicine, Maine Medical Center, Portland, ME, United States

**Keywords:** COVID-19 testing, structural vulnerability, qualitative, access barriers, health behavior model

## Abstract

**Background:**

People with structural vulnerabilities (including immigrants, people who use drugs, and those who are unhoused or uninsured) are more likely to experience COVID-19 testing disparities relative to other groups. We documented barriers and facilitators to COVID-19 testing and explored how structural vulnerabilities created and/or exacerbated COVID-19 testing barriers.

**Methods:**

Between 2021 and 2022, we conducted semi-structured interviews with 34 members of structurally vulnerable populations and 27 key informants who provide health and social services to them. Our abductive analysis was iterative, utilizing both inductive and deductive coding processes. Recognizing that adequate and appropriate testing for COVID-19 is a complex health behavior that involves both decision-making and issues related to access, we developed a hybrid model of COVID-19 testing behavior to organize reported barriers. We then used that model for more in-depth analysis of structural vulnerabilities in the context of testing.

**Results:**

Our model of testing behaviors provides a framework for understanding the many barriers and facilitators relevant to COVID-19 testing. After identifying locally-reported barriers, we found that specific conditions—economic precarity, legal precarity, the confusing U.S. healthcare landscape, English-exclusive environments, and stigmatizing medical encounters—make adequate and appropriate testing less likely by making COVID-19 testing feel riskier (entailing legal, financial, and psycho-social risks) and making healthcare, and thus vicariously testing, more difficult to access.

**Conclusion:**

The COVID-19 pandemic exposed disparities in health care delivery. To avoid under-testing and its associated health consequences during the next pandemic, public health efforts should address structural conditions to ameliorate risks and bolster testing infrastructure to improve access.

## 1 Introduction

During the COVID-19 pandemic, SARS-CoV-2 testing was necessary to track infections, prompt isolation, and refer for treatment. However, populations most at-risk for infection, including Black and Latino individuals (1, 2) and people in poverty (3) or experiencing homelessness (4), faced barriers to COVID-19 testing (5, 6). The lower testing rates among at-risk populations led to the under-detection of infections in communities where controlling outbreaks were most important (7). Barriers to adequate and appropriate testing likely also played a role in the disproportionate affect the COVID-19 pandemic had on health outcomes for at-risk populations, including higher rates of hospitalization and death (8).

Past research has identified many different barriers to COVID-19 testing experienced by various populations in the US from 2020 to 2022. Barriers to accessing COVID-19 tests included: cost of testing, transportation issues to testing sites, lack of interpreter services, low health literacy around the purpose of testing, poor communication, confusion about how to test, and misinformation (9–13). Studies have also reported a variety of reasons as to why people decide not to get tested, such as: concerns about testing accuracy, physical discomfort caused by testing (i.e., nasopharyngeal swab method), exposure to others with COVID-19 at testing sites, concern of infecting others, misuse of personal information collected at the time of testing, and impacts of testing positive such as stigma and loss of resources (10–12, 14).

Although many studies to date document barriers to COVID-19 testing, few studies examine how the *structural vulnerabilities* faced by at-risk populations may exacerbate these barriers. Structural vulnerabilities encompass the socioeconomic, political, and cultural power hierarchies/systems that hinder the ability for certain groups to engage in healthcare and, consequently, put them at greater risk for poorer health outcomes (15). To deliver assistance where most needed, public health efforts can focus on addressing structural vulnerabilities, based in systemic issues rather than individual-level deficits (16). As such, there is a need to understand the ways in which structural vulnerabilities impact COVID-19 testing behavior to better inform public health strategies for future disease outbreaks and improve health equity.

The National Institutes of Health (NIH) established a funding initiative called Rapid Acceleration of Diagnostics – Underserved Populations (“RADx-UP”) to support community-engaged research to improve COVID-19 testing among underserved populations throughout the US (17). The purpose of our RADx-UP study was to identify and reduce testing barriers for structurally vulnerable populations in Portland, Maine, specifically: people experiencing homelessness, immigrants, and people who are low-income/uninsured. In this article, we present the findings from the qualitative component of our larger RADx-UP study. The research aims of the qualitative component were two-fold: (1) to uncover barriers to COVID-19 testing for those specific populations and (2) to explore how structural vulnerabilities create and/or exacerbate the testing barriers.

## 2 Materials and methods

### 2.1 Design

We worked in partnership with community-based organizations (CBOs), who supported recruitment and provided feedback throughout the research process. The CBOs we partnered with included: (1) Preble Street, an organization providing social services to people experiencing homelessness as well as healthcare through a collaboration with the local hospital (authors CF and AV); (2) Greater Portland Health, a federally qualified health center with a high proportion of immigrant patients (author AT); and (3) the Portland Community Free Clinic, an organization that provides free medical care to uninsured adults (author LN). Given the complexity of testing during this time period, we used semi-structured interviews to permit discovery and exploration of unexpected information. We interviewed both community members—belonging to structurally vulnerable populations—and key informants—people providing health and social services to these populations—to collect individual and system-level perspectives on testing challenges, respectively. We employed an abductive approach to analysis, which was both deductive and inductive, using theoretical models to reveal and explore discrepant data (18). Our study protocol was considered of minimal risk and exempted from review by the MaineHealth Institutional Review Board.

### 2.2 Populations studied

We used heterogeneous purposive sampling of community members and key informants. Purposive sampling allowed us to focus on identifying cases with the most relevant information on barriers, specifically people likely to experience structural vulnerabilities—people experiencing homelessness; immigrants, refugees, and asylum seekers; people who inject drugs, and other people accessing city services (Portland Community Free Clinic, STD Clinic, and Portland Needle Exchange)—and people who provide services to these populations, including community health workers (CHWs), medical interpreters, and other staff. We drew a heterogeneous sampling of different groups to understand how particular characteristics of the social, political and economic environment broadly impacted structurally vulnerable populations (these groups are identified in Table 1). We sought a minimum of four community members and two key informants from each target population noted above. For immigrants, refugees and asylum seekers, we included 6 international populations, representing the most common languages spoken by patients accessing Greater Portland Health—Arabic, Spanish, Somali, French, Lingala, and Kinyarwanda (personal communication on September 30, 2021 with the Chief Medical Officer at Greater Public Health). Our team included interviewers fluent in Spanish, Arabic, Somali, Lingala, and French. Interviewers also had access to telephone interpreter services.

**Table 1:** Community member participant demographics (*N* = 34).

Participant characteristics	*N*	%
Mean age in years (SD)	41.97	(12.77)
Gender identity*		
Female	17	50%
Male	16	47%
Race or ethnic identity*		
Black	15	44%
White	16	47%
Hispanic	1	3%
Other	2	6%
Population group		
International	16	47%
Latina/Latino	1	
Arabic speakers (Iraqi/Syrian)	2	
Somali	2	
Burundi/Rwandan	3	
Congolese	4	
Angolan	4	
Unhoused	8	24%
Needle Exchange clients	2	6%
Low income/uninsured	4	12%
STD clinic clients	4	12%
COVID characteristics		
Vaccination status		
Vaccinated and boosted	9	26%
Vaccinated, but not boosted	17	50%
Unvaccinated	8	24%
Taken a COVID test before	28	82%
Knows someone who had COVID	23	68%

*Self-ascribed.

### 2.3 Data collection

In cooperation with our community partners, we developed semi-structured interview guides to identify barriers to testing and inform interventions. Interviews took place from October 2021 to April 2022, with all but seven interviews completed before February 2022. We restricted recruitment to this short period because the course of the pandemic was shifting rapidly, including greater availability for at-home testing and relaxing CDC guidelines.

Community partners, who were trusted by local communities, endorsed the study and assisted with recruitment of community members for participant interviews by displaying flyers and directly telling patients/clients when interviewers were on site at the CBO. Members of the study team, including medical students and student-researchers, were trained in conducting the interviews and ensuring community members were comfortable sharing candid information. Community members were asked about how the COVID-19 pandemic had impacted them, knowledge and beliefs about COVID-19, and experiences with COVID-19 testing including any challenges with testing and reasons not to test. Feedback from early interviews was used to modify the guide.

Key informants were identified by community partners, other community-based organizations who were also aware of the study, and other key informants. They were recruited through direct email by the interviewer. All key informant interviews were conducted by experienced interviewers who elicited narratives about broader issues of COVID-19 testing in these communities based on what key informants had seen or heard from their clients/patients. Interviews specifically probed into challenges, barriers and facilitators to testing for the respective population. Because key informants worked in a variety of settings with different populations, interviewers exercised flexibility in tailoring questions to ensure the most relevant information was obtained.

Community members and key informants provided informed consent prior to interviews, and no identifiers (e.g., names) were collected. Community members were compensated with a $20 grocery gift card. Because community partner organizations were directly compensated, key informants working for partner CBOs did not receive additional compensation, but key informants working for other CBOs received the same gift card as community members. Interviews were audio recorded and professionally transcribed. Interviews in languages other than English were translated and transcribed by fluent interviewers into English.

### 2.4 Analysis

Our approach to analysis was abductive, involving iterative cycles of inductively developing theories or models, which were then used deductively to make sense of data, with attention to discrepancies that give rise to new or revised understandings (18). We began our analysis by uploading interview transcripts into MAXQDA (Version 22.8.0) and coded the transcripts using Iterative Categorization (IC), a systematic coding approach that begins by segmenting transcripts based on question or interview topics and proceeds by inductively sub-coding those segments (19). Specifically, three analysts coded transcripts based on topics covered in the interview guides: impacts of COVID-19, testing experiences, decisions related to testing, and anything reported to make testing more difficult or less likely (barriers), including reasons not to test when testing could be considered appropriate. Barriers were conceptualized broadly to ensure sensitivity to issues that may be less obvious. Topics were not mutually exclusive, so analysts were able to assign multiple codes to the same segments. During coding, we familiarized ourselves with the data while honing sensitivity to cross-cutting themes (20). Among these themes were references to the spatiotemporal context in which people were getting tested. We narratively summarized this context and included it as background for subsequent analysis.

To address the first research aim, we focused on identifying barriers to COVID-19 testing. The two lead authors independently reviewed transcript excerpts previously coded as barriers, and inductively sub-coded specific barriers. We each developed alternative frameworks for organizing these barriers, and then met to compare and discuss. We noted that some of our barriers seemed to fit Penchansky, Thomas and Saurman’s model of access, including accessibility, affordability, acceptability, awareness, accommodation, and availability (21, 22). However, other barriers we identified—concerning decisions about whether to test—were not clear in the model. Recognizing the complexity of barriers to appropriate and adequate testing, we began to develop a hybrid model that merged this model of access with the RANAS (Risks, Attitudes, Norms, Ability, Self-regulation) model of behavior change (23). We then reviewed testing experiences reported in the interviews to understand how both decisions about testing and issues of access intersect to create testing barriers, revising our model to mirror these experiences. This model is described in our Results.

To address the second research aim, we examined overlap between structural vulnerabilities and testing behavior, using our model of COVID-19 testing behavior to conceptualize how vulnerabilities exacerbated specific testing barriers. In continuance of our abductive approach, we considered which aspects of testing were left unexplored by our model. Drawing on our memos and theming from earlier stages of analysis, we identified specific structural conditions referenced by community members and key informants. Using these structural vulnerabilities as a lens, we revisited our previously identified barriers and excerpts coded as “testing experiences” to consider how particular structural conditions exacerbated testing disparities.

To ensure trustworthiness of results, we iteratively reviewed our claims to ensure they accurately reflected statements from community members and key informants, and we triangulated on-the-ground understandings of testing access by comparing reports from different groups. Within the analytical team, we employed the “critical friends” approach wherein team members were expected to challenge the interpretations and claims of their fellow analysts (24). We also presented preliminary interpretations and findings to our community partners to invite feedback and ensure themes resonated with their experience.

## 3 Results

We interviewed 34 community members, whose demographics are summarized in Table 1. Though we met or exceeded our goals for most groups, we were unable to meet base recruitment goals for Latino/as, Iraqis, Syrians, and Somalians. Community member interviews took 10–52 min, and typically took place at CBO sites while these participants were accessing care. We conducted 27 interviews with key informants, whose characteristics are summarized in Table 2. These interviews were virtual, taking 15–60 min.

**Table 2:** Key informant participant characteristics (*N* = 27).

Informant characteristics	*N*	SD/%
Mean years of experience (SD)	6.6	(4.96)
Type of role		
Leader of CBO serving immigrant community	1	4%
Community Health Worker*	6	22%
Medical Interpreter+	6	22%
Nurse (Portland Community Free Clinic)	2	7%
Public service worker (STD clinic, Needle Exchange, Preble Street, Greater Portland Health)±	12	45%
Population group most engaged with		
Immigrants and refugees	13	48%
Latino/Latina	3	
Arabic speakers (Iraqi/Syrian)	2	
Somali	1	
Burundi/Rwandan	1	
Congolese	2	
Angolan	1	
Multiple	3	
Unhoused	6	22%
Needle Exchange clients	3	11%
Free Clinic clients	3	11%
STD clinic clients	2	7%

*CHWs were employed by the local hospital, city health program, or a CBO serving the immigrant community. +Most medical interpreters were employed by the local hospital. ±Public service workers held positions such as patient care navigator, health guide, social worker, outreach worker, program coordinator, program director, and more.

To ensure generalizability, our results go past locally encountered barriers to broader patterns. We begin with context for COVID-19 testing during this period in Portland, Maine based on key informant interviews. Next, we summarize our COVID-19 testing model, which we used to organize barriers and facilitators. Finally, we describe how structural vulnerabilities intersect the model, making certain barriers more formidable in these populations. Representative quotes ground each claim. Unless otherwise noted, all claims are based on reports from multiple populations of interest. Ethnic identities included in quote attributions are based on how community members self-identified. Key informants are identified based on vulnerable group with whom they work.

### 3.1 Context: COVID-19 pandemic, 2021–2022

When our interviews took place, in late 2021 to early 2022, the COVID-19 pandemic had been going on for about two years and COVID-19 tests had been in use for more than a year (25). During this period, the U.S. entered the Omicron variant surge, and the first COVID-19 vaccine booster shot was recommended (25). In Maine, booster shots had just started to become available, but the rate of initial COVID-19 vaccination was relatively high at 70.8% (26). With regards to testing, in the greater Portland area several CBOs provided free COVID-19 testing and there were a few high-volume public testing sites, such as at the local airport. As part of our larger RADx-UP study, our team ran low-barrier walk-up testing clinics at our community partners’ sites starting in January 2022 (27). The Maine CDC’s Office of Population Health Equity had also established a “community care referral form” to connect vulnerable people sick with COVID-19 to social services, such as food and transportation, via community organizations throughout the state (28).

Although these testing resources existed, at the time of our interviews there were several global and local circumstances that created challenges relevant to testing. The State of Maine had COVID-19 restrictions in place to limit exposure of infected. Testing positive for COVID-19 in Maine in late 2021 required isolating from other people for at least a week: children were asked to remain at home rather than go to school or daycare, and adult caregivers could not work (29). Key informants emphasized (see Table 3 for supporting quotes) how gaps in the healthcare delivery system that existed prior to the pandemic were exacerbated as more people needed services while fewer services were available (30). COVID-19 policies restricted access to healthcare facilities or temporarily closed sites providing resources. For example, shelters restricted new occupants following an outbreak of COVID-19, limiting overall shelter availability. One clinic, which provided healthcare access to many international patients, could only provide access to established patients of the clinic.

**Table 3:** Key quotes about COVID-19 pandemic context, late 2021-early 2022.

Topic	Excerpt	Excerpt
Gaps in healthcare delivery	…the hardest part is getting people into any sort of [substance use] treatment. Treatments can be closed due to COVID, not having enough staff. So getting people options into treatment is… There’s just none available (Informant 2, Syringe Service Provider)	…since there’s been a shortage of tests, [community clinic A] will only test patients that are already established in their clinic. And so we cannot send over folks that are not [community clinic A] patients. So that means we have to schedule an appointment [with Maine Medical Center] (Informant 13, Social Worker, Unhoused)
Lack of information	…that was most of the questions that we would get is, “where do I go get tested? How can I get tested? I do not have insurance. How do I do this? Where can I go? How’s the turnaround? Do I need to make an appointment?” So even though, and I know tons of that information was out there, and typically as things would change, we would try to stay in the know to the best of our ability to help people out when they called. But I felt like it was just an ever-changing situation (Informant 1, clinic coordinator, STD clinic)	And so to be able to collect those resources for the staff that I supervise, it’s like I’m coming up with nothing too. And so I’m using the information that I have from my experience working at the quarantine shelter, that’s super helpful and great, but if I’m not at work and someone is like, “I do not have the answer to this.” It’s like no we can always call the quarantine shelter and those staff and speak to the clients and stuff. But it’s not great (Informant 12, shelter supervisor)
Confusing information	There’s just a lot of… And I know this has been out there, but there’s a lot of misinformation and just getting… We’re now at the point of trying to vaccinate, the reluctance… They just do not have faith in what the FDA or the CDC says about these vaccines (Informant 3, Nurse, Low-income/uninsured)	She said, “no, I would not get tested. I do not really believe in all this stuff.” And she cited all the mixed messaging that was happening since the beginning, “First you should not be wearing masks, because they are do you any good, and then everybody should mask. And then nowadays people are more and more vaccinated, but they still have to wear these masks and we can still get sick, even if we are vaccinated and nothing’s changed even with the vaccination.” So she was just really suspicious about the conflicts and the contradictions and what she is seeing is happening (Informant 17, Interpreter, Portuguese and French)

Furthermore, an evolving situation produced a dearth of accurate information about changes to testing policies, COVID-19 restrictions, and outbreak-driven closures of testing sites. Even information about the tests themselves, such as possibility of false positives or negatives, was changing to reflect latest data. Information communicated via websites and social media required individuals to have an electronic device, internet access and knowledge of which platforms had most up-to-date information.

Meanwhile, as documented elsewhere (31, 32), misinformation sowed confusion and concerns around COVID-19, vaccines, and tests. Community members observed the spread of this misinformation in their own communities, some reporting family members skeptical of COVID-19. Key informants suggested misinformation bred reluctance to accept medical advice in communities.

### 3.2 Model of COVID-19 testing behavior

**Figure 1:**
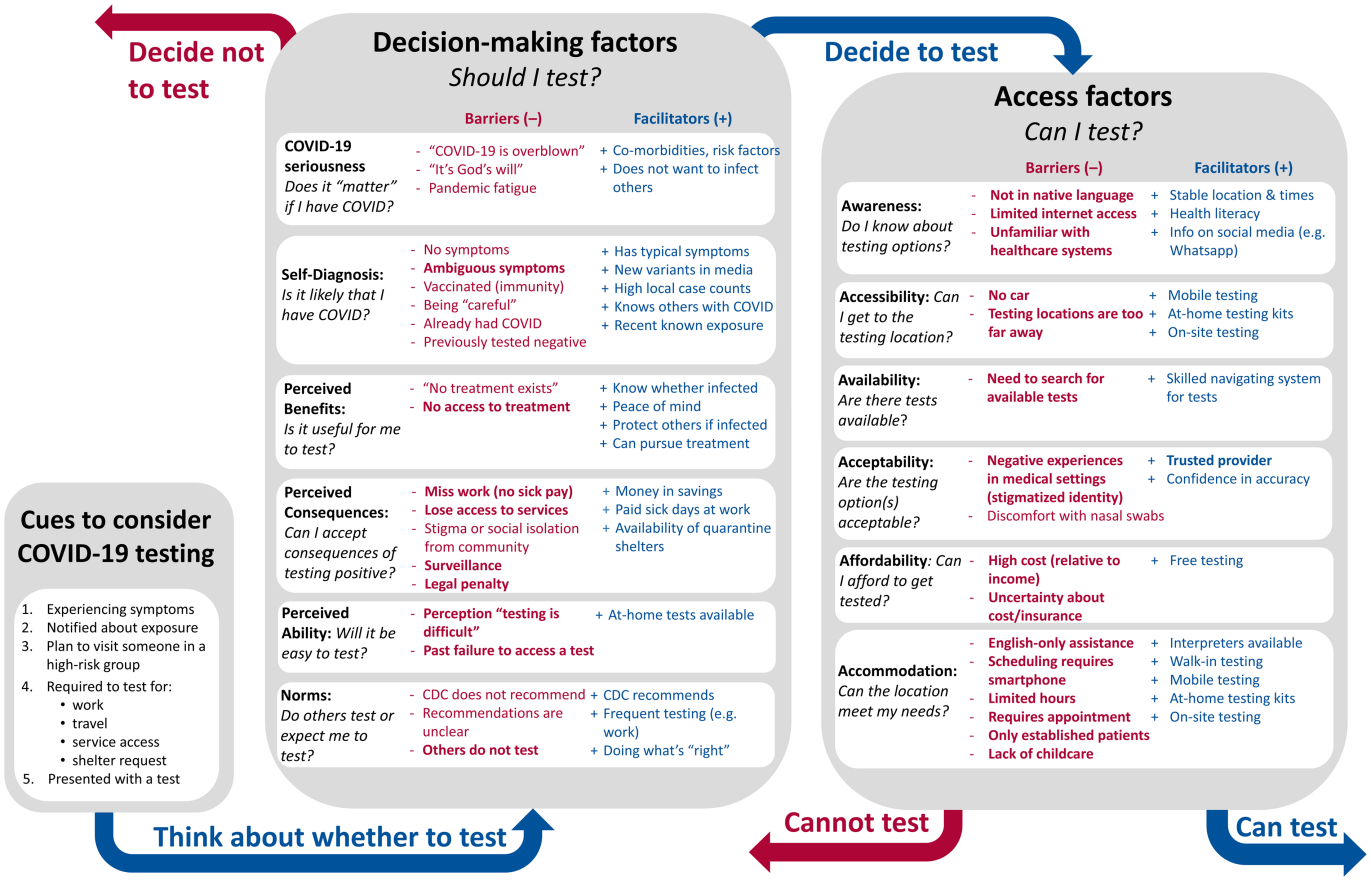
Descriptive Model of COVID-19 Testing Behavior. Facilitators (blue) and barriers (red) were reported in Portland, Maine 2021–22, and are organized based on which aspect of testing they impact. Barriers closely associated with structural vulnerabilities are bolded.

Our analysis of testing barriers led to the development of a descriptive model of COVID-19 testing behavior that captures the full scope of barriers and facilitators identified in the interviews (see Figure 1). The model recognizes three conditions required for appropriate and adequate testing. First, an individual must be cued to test. The cues we heard from our community members and informants included the experience of symptoms, learning a contact tested positive, a requirement to test (e.g., for work, school or to access services), a planned visit to someone in a high-risk group, or even the presence of a test:

They only get tested if they suspect and/or they are required to test because of their work. I think generally people do not want to get tested unless there’s a reason. And the reason is not necessarily symptoms. The reason is usually, like, I’m required to by someone else (Informant 24, CHW, Multiple immigrant groups).

Next, an individual must *decide* whether to test (i.e., Should I test?). This decision involves several considerations—of whether they believe COVID-19 is important (seriousness), of whether they think infection is likely (self-diagnosis), of whether they believe there is any benefit in getting tested (perceived benefit), of what negative consequences could follow testing (perceived consequences), of how difficult they imagine it would be to get tested (perceived ability), and of whether they believe others would expect them to test (social norms). Various facilitators and barriers impacted this decision, and these were organized by the relevant consideration. For example, the belief that COVID-19 vaccines granted immunity to infection meant that many vaccinated people decided not to test because they believed infection was unlikely or impossible (i.e., self-diagnosis).

Finally, individuals who decide testing is desirable, must be *able* to test (i.e., Can I test?), which requires all six dimensions of access: awareness of how and where to access tests, logistical accessibility of tests, availability of tests, affordability of tests, acceptability of testing options, and accommodations offered at those testing locations. Again, different barriers and facilitators were relevant to different dimensions of access, as shown in Figure 1.

Whereas Figure 1 suggests linearity, we recognize that decisions about testing and testing access are integrated in practice. Expectations about the difficulty of testing are a factor in decision-making, and past experiences of testing barriers may make deciding to test less likely. Furthermore, individuals who are strongly convinced that testing is necessary may assign it a higher priority and expend more material and social resources to access tests than someone who is uncertain about whether to test. While a person’s circumstances for testing may obviate or complicate steps in the model (e.g., if tests are provided directly), organizing the barriers to appropriate and adequate testing can reveal broader patterns. All facilitators and barriers included in Figure 1 were mentioned in multiple interviews.

### 3.3 How structural vulnerabilities impact COVID-19 testing behavior

We identified certain conditions that exacerbated barriers to testing, disproportionally impacting structurally vulnerable people. These conditions included economic precarity, legal precarity, the confusing U.S. healthcare landscape, English-exclusive environments, and stigmatizing medical encounters. Table 4 shows whether each of these conditions were mentioned by community members or key informants from each of the populations of focus. We caution that we did not systematically ask about these conditions during interviews, so the fact that a condition was not reported for a group does not mean it was not relevant, only that it was not spontaneously mentioned. For example, clients of the needle exchange likely also encountered a confusing healthcare landscape, but it was not mentioned in interviews as relevant to testing.

**Table 4:** Structural conditions described in the interviews for each vulnerable population.

	Unhoused	Immigrant	Free clinic	STD clinic	Needle exchange
Precarity, economic		x	x	x	
Precarity, legal	x	x			x
English-exclusive environment		x			
Stigmatizing medical encounters	x	x	x	x	x
Unfamiliar and confusing healthcare landscape	x	x	x	x	

Specific decision-making and access barriers linked to structural vulnerabilities are bolded in Figure 1. As can be seen, structural vulnerabilities particularly exacerbate issues related to access (Can I test?), relevant across all six dimensions. Many impacts on decision-making (Should I test?) also relate to healthcare inaccessibility. For example, if individuals have encountered access barriers in the past, those experiences will shape their Perceived Ability to test. Healthcare access also impacts Perceived Benefit of testing if people do not believe they will be able to access treatment even if positive. Beyond access, however, these structural conditions worsened the Perceived Consequences, raising a variety of legal, financial, and psychosocial risks, or undermining ability to assess those risks. In the following sub-sections, we describe how each condition impacts decisions and access.

#### 3.3.1 Economic precarity

We use the term economic precarity to refer to a state of living “paycheck-to-paycheck” such that unexpected expenses risk inability to pay for basic needs such as food and shelter.^25^ Aside from unhoused persons, who experience a more extreme situation than mere precarity, all of the populations of focus experienced economic precarity to an extent. For people experiencing economic precarity, testing is financially risky. The costs and consequences of testing are relatively higher for a person experiencing economic precarity than for someone with a reliable income:

They have some resources, but they think carefully about, “Well, can I afford this? If I have to choose between this and that, how am I going to think about my choice?” And I think that just living that lifestyle, they just become frugal (Informant 5, Nurse, Low-income residents).

One financial risk of testing involved paying for the test. In the US, COVID-19 testing was not always free, and in some contexts (e.g., emergency rooms, urgent care clinics, etc.) the cost of testing is not clear. Individuals understandably prioritize meeting basic daily needs—like food, medicine or shelter—over testing. Accordingly, when seeking options for testing, community members were reluctant to seek out testing that was not free:

I mean, I could have probably paid money, but I really did not feel like that was a viable option as far as what I was looking towards. I was scared that I just did not have the money, so yeah, I wanted a free test… (Participant 19, Public service-user).

A much greater financial risk came with the possibility of testing positive for COVID-19 and needing to quarantine, miss work, and lose income or other resources.

[Many recent immigrants] do not have a stable job. They do not have any financial stability. They are still trying. They’re building everything from scratch. […] If you see the way they are increasing their rent in Maine, someone with that job without working for 10 days, that will affect the person. […] People have to pay the rent, insurance. They’ve got kids. They have to take care of everyone. This is to say one on one hand, [COVID-19 isolation policies] protect the person, on the other side, it’s like killing the person also. […] This is to say, they may refuse to identify as infected. […] They will try to refuse to stay home. They’ll try their best because they have got bills (Participant 27, Congolese).

…in this country, a lot of people who live here are living paycheck to paycheck. Staying for a week or two weeks at home makes a big difference when it comes to how much money they make. The idea of having to stop working had a lot of concern. And when you think about the jobs that a lot of our communities have, they do not have sick time or vacation time (Informant 20, CHW, Arabic residents).

I pray and ask God not to get COVID because I need to pay a rent (Participant 8, Somali).

For people whose insurance is contingent on employment, missing work could mean also losing healthcare coverage:

I had a chance of having [a primary care physician], but with the COVID, I had a problem with my insurance because I could not pay it. They put me in quarantine and I was not working, and they told me that if you spend a month without working, you have to renew it. So I’ve lost everything. I could not renew it. I did not have money, and it was also very expensive (Participant 30, Burundian).

The financial impact of loss of income was especially felt by multi-generational immigrant households where many family members rely on a single person’s earnings:

It is the case that at the start of the pandemic, they had families to take care of, most of them. Not being able to provide for them was something that they could not have afforded (Participant 3, Honduran).

In these situations, many described choosing economic obligations over health:

Yep, but to be honest I was sick for a week, but I went to work. It was really bad. I felt I was taking a risk all the way, but I had to because of work and I needed the money. Gets tough sometimes (Participant 5, Arabic).

The financial strain of testing positive shifted throughout the course of the pandemic. Early in the pandemic the financial hit was harder because of the lack of social supports, and long isolation period of 10 days. When financial assistance for food and housing was available, people became more willing to test:

…at first, they did not even actually have any agency to provide food […]. So it was a time when people did not want to take test then, because the results was worse than having it, than go with it. […] Then after, when Opportunity Alliance was started to pay for houses, for rent, other organizations, started to pay for food. People were actually more than welcome, actually was welcome in this test, to be tested (Informant 23, CHW, Central African residents).

If someone experiencing economic precarity chooses to test, they may still face numerous access barriers. Many such barriers can be overcome with resources, including transportation, childcare, smartphones, internet access, insurance, and ability to pay. However, people experiencing economic precarity, or the possible result of precarity—homelessness—have fewer resources to overcome access barriers. The condition tends to limit transportation options, which impacts ability to access COVID-19 testing. This was especially true for people experiencing homelessness and newly-arrived asylum-seekers:

…transportation is a big issue, especially now when the shelters are overflowing and so many people are living in South Portland in the hotels and motels over there. To get to a testing site is difficult. Especially if you got bunch of kids and how are you going to get- putting everybody on a bus and going to a testing site? (Informant 24, CBO Leader, Refugees/Immigrants).

Some testing locations were difficult to access by foot or public transportation. If a testing site closed unexpectedly, transportation costs multiplied. Finally, people needed internet access and smartphones to find up-to-date information on testing locations, to schedule testing appointments and to access results:

…a lot of tests that you have to access a patient portal or get a text to your phone, or if you receive your status digitally, that’s another issue. ‘Cause a lot of people do not have access to consistent internet and consistent avenues to get those testing results (Informant 8, Outreach Worker, Unhoused residents).

#### 3.3.2 Legal precarity

Structurally vulnerable people may also be *legally precarious,* wherein gaining the attention of law enforcement could have legal consequences. Legal precarity impacts anyone participating in the informal economy, including some undocumented immigrants and people experiencing homelessness:

…If they are undocumented, there’s always the risk that if I give my name and date of birth, what does that mean? Who’s tracking me? Who’s sharing this information, and with whom, and does it mean I’ll get picked up and deported? (Informant 9, Spanish Interpreter).

Similar to economic precarity, the condition of legal precarity shaped decisions to test because, especially when testing positive, it meant that testing could bring legal risks—potential surveillance or legal recourse, which could put the asylum process in jeopardy. While many refugees said they would obey CDC requests for testing, some suggested legal fears drive reluctance:

…lots of people are saying that the CDC is like the police. They’re a type of police that’s telling everyone who’s sick and that scares people. That’s why people avoid them sometimes (Participant 29, Angolan).

Among unhoused populations, informants similarly noted that some people refused to provide personal information for testing:

A lot of folks like to fly under the radar. And sometimes it’s a struggle for people who are new to this community to even want to give us their real names. So I do think that if people were informed that it would be reported to the CDC, that could be a deterrent for some people (Informant 6, Health guide, Unhoused/Low-income residents).

#### 3.3.3 Unfamiliar and confusing US healthcare landscape

Many informants suggested that immigrant community members are accustomed to healthcare systems in their country of origin and as a result have different expectations when navigating unfamiliar US healthcare systems. This cultural difference in expectation, particularly around *when* to engage with healthcare, impacts decisions around testing.

Key informants explained that, where their clients are from, people only access doctors to obtain medicine for disease treatment (i.e., when a person is actively sick), and do not engage with healthcare for reasons of preventive medicine. As a result, people may not decide to test in the absence of symptoms due to these norms of healthcare use.

…culturally, that these communities, they use healthcare when they need by themselves, so when given, when advised to take, or when they are told that, “you have to test even though you do not have any symptoms,” some of them will not accept immediately the first time… (Participant 8, Somali).

Even when symptoms are apparent, community members reported that members from their immigrant community may decide not to test because there is no treatment available and thus no perceived benefit to testing or engaging with healthcare.

Some people may say that it’s not good to get tested because the disease itself cannot be healed by the medical doctor (Participant 4, Congolese).

Confusion around how the US healthcare system works, including when and where to receive services, also influenced testing. Many community members from African countries said they believed the hospital was the appropriate place to be tested for COVID-19, even when outpatient options were available. This confusion impacts access, as people are unaware about potentially more convenient testing locations, as well as decision-making because of greater perceived consequences of testing. Some immigrant community members anticipated that going to the hospital for testing risked being forced to quarantine, including separation from family members, and thus home treatment was preferable:

…someone might go to the hospital because they are having difficulty breathing. They’d go to the hospital in that case. But if their breathing is normal and they do not want to be isolated or quarantined [if they test positive], they’ll stay home. Because at home, there are people there to help you (Participant 29, Angolan).

Uncertainty about the financial aspects of the US healthcare system, especially when it comes to insurance and medical care costs, makes it difficult to assess the financial risks of testing and receiving an unaffordable bill. When costs were not communicated, some people avoided testing or other medical care, even in cases where testing was actually free or affordable:

…some, they do not want to seek for treatment or to go to see a doctor because they say, “I do not have insurance. How can I pay my bills?” They are struggling about that and then they do not want to see a doctor or to seek help when they are sick, because they think that it’s like in Africa, you need to pay first before they can see you. […] Some, they do have even insurance from their work, but they do not know how it works here. They will say, “I heard even when you have insurance, you have to pay.” So they do not know the system, how it works and they make decision[s] according to what they hear because they do not have much information how the system works here (Informant 21, CHW, Congolese/Angolan residents).

Even if someone who was still learning about the US healthcare system decided to test, the confusing system creates additional barriers to accessing them, including awareness around where and how to test. One participant recounted successfully accessing tests, but only because she had a car, internet access and ability to navigate the system:

I would go online and usually the one nearest me was out. There was never… I was never usually lucky enough to have one nearby […] I got one in Saco [17 miles away]. […] I found out if I typed into Google, “At-home COVID test,” then I clicked on the Walgreens link that came up specifically for at-home COVID test, they would say the nearest location and it would be probably “not available.” And then it would allow you to click something; if you figured it out to scroll down, you would see: “In stock.” (Participant 19, Public service-user).

Logically, people unfamiliar with systems of care lack knowledge to navigate it effectively. Though the confusing nature of US healthcare was most apparent for immigrant populations, similar issues of navigation impact anyone attempting to access tests.

#### 3.3.4 English exclusive environments

Another layer of complications arises for people with limited English proficiency navigating environments without linguistic accommodations. English exclusive environments impact both decisions about testing—including added uncertainty associated with interpretation—and access. As one medical interpreter explained, their clients have the perception that it will be too difficult to get tested because of the anticipated language barriers and thus decide not to test:

But the language barrier is a huge obstacle. And I think that if people have to call up on their own and not know if they’d get an interpreter on the line to get information about testing, they would not do it. I mean, it is a large barrier (Informant 19, Spanish Interpreter).

When a person does decide to test, several informants and community members described how difficult it can be to figure out how to access a COVID-19 test, as it can require many logistics. When testing resources are only available in English, this creates an additional barrier to access for non-English speakers especially issues of awareness around where and how to access a test.

Do I have to make an appointment? So for me as an English speaker, living in a community, working in a hospital, it would be an inconvenience. So again, we are adding on logistical barriers for our community, language barriers, and misinformation (Informant 7, Arabic Interpreter).

Since public health messaging around COVID-19 and testing is often only provided in English, this information cannot be accessed by immigrant communities with limited English proficiency. Key informants reported that some people in immigrant communities instead rely on social media and news sources from their home country, leading to misinformation and a lack of locally relevant information.

I think there’s a lot of fear, a lot of confusion, a lot of misinformation, similar to any community in general population, except there’s the added barrier of language. So announcements, say, from the CDC, aren’t getting to our communities. […] A lot of these communities tend to have satellite dishes in their homes where they receive the news from their home countries. So they are not so involved with the news in the U.S., or locally at all (Informant 7, Arabic Interpreter).

#### 3.3.5 Stigmatizing experiences and distrust of medical facilities

Some community members avoid testing sites, reporting uncertainty about how well they will be treated if COVID-19 positive. Many in our populations of focus have stigmatized identities: “immigrant,” “homeless,” “poor,” or “drug-addicted.” Some people who are unhoused or inject drugs have had negative experiences, including stigmatizing encounters and/or medical trauma, and fear medical sites as presenting psychological risk of further stigmatization. As such, these past negative experiences create issues of access because medical sites become unacceptable locations for COVID-19 testing.

…they feel like they are constantly being insulted, constantly being degraded and demeaned. I mean, I’ve seen it firsthand, especially doctors, first responders, kind of universally. So a lot of them have just had so many bad experiences that they are like, ‘I do not even want to deal with that. I’d rather get COVID and get through it then have to deal with a doctor.’ (Informant 8, Outreach worker, Unhoused residents).

Accordingly, key informants said that community members preferred to access medical care, including testing, from providers and clinics that they trusted based on a record of positive interactions. In other words, trusted providers are a facilitator for access by making the testing experience more acceptable.

If that role [recommending testing] is taken by someone they trust like us as community health workers, or community leaders, or faith leaders […] then it’s a possibility that people will accept that (Informant 15, CHW, Somali residents).

Notably, we heard more about distrust of providers for people experiencing homelessness and less often for refugees and immigrants:

There are people understanding and there are people who are respecting the providers. […] In my country, we are respecting the providers because they are like a, I can say, a small god, because they do have your life, so we are believing in them. Whatever they prescribing, we are taking the medication. Yeah. They cannot say no, they are respecting whoever advises (Informant 25, CHW, Rwandan/Burundian residents).

However, unfamiliar experiences interpreted through cultural expectations can be misunderstood, impacting trust:

The trust is there, but lack of trust is [too]. Because sometimes it’s from the doctor himself, and sometimes from the client because they do not know how the system works […] [interpreters] are compelled to just facilitate the communication and tell what the patient is telling and what the doctor telling, and the time is limited. That’s why they sometimes people say, “We are not getting what we want to hear.” They want to hear stories. They want to tell everything they have, but sometimes there are no time to listen (Informant 15, CHW, Somali residents).

Inability to pay for medical care was also a source of fears about stigmatization, which also made visiting medical facilities psychologically risky:

…the people we see are out of the healthcare system, without health insurance […] they are only accessing healthcare through the Emergency Room because they know they have to be seen, and then they end up owing thousands of dollars in healthcare costs that they have never been able to pay. So they stay away from the hospital because they think the next time that they are sick, that they are going to be somehow nabbed for… The computer’s going to bring up all these past bills, so they do not go (Informant 3, Nurse, Low-income/Uninsured residents).

## 4 Discussion

We identified barriers to adequate and appropriate COVID-19 testing and developed a model to organize associated barriers and facilitators. Then we examined how structural vulnerability intersects with COVID-19 testing behavior, including both decision-making and access to testing. After identifying five structural conditions—economic precarity, legal precarity, a confusing healthcare landscape, English-exclusive environments, and stigmatizing medical experiences—we examined how these conditions created or exacerbated testing barriers. We found that these conditions restricted adequate and appropriate testing for several reasons. First, they dis-incentivize COVID-19 testing by creating financial, legal, psychological and social risks associated with testing. Testing may invite financial strain through hidden medical costs and loss-of-income, and testing surveillance may invite legal risks for some community members. Furthermore, some consequences of testing include social risks, including isolation, and psychological risks involved with interacting with stigmatizing medical systems. Second, they undermine access to medical care, which further limits access to tests and undercuts a perceived benefit of testing—medical treatment if positive.

The barriers we identified in our study are consistent with those identified in other settings, including among other structurally vulnerable populations, impacted by precarity, stigma and unfamiliarity with the health system (10–13, 33). For example, Lee and colleagues noted the role of weak safety nets, among issues like inaccessible testing sites and lack of testing supplies and staff, as barriers for some communities (33). Other researchers have reported that anticipated stigma from medical personnel make some people less likely to test (34), and that adverse experiences, mental health disorders, and legal troubles spawn fears about sharing information (35). One community based participatory research study among underserved Latino communities identified similar barriers—mistrust, job/income loss and stigmatization—though they conceptualized them as “personal” rather than structural (11).

The healthcare delivery system in the US is confusing even for most citizens—obtaining coverage, identifying providers covered under insurance plans, and uncertainty about out-of-pocket costs demand resources and create financial risks around engaging systems of care (36). People emigrating from outside the US face additional challenges navigating this system, especially with limited English proficiency. Expectations about engaging the healthcare system are partially based on healthcare experiences in other countries. When people attempt to engage, but their expectations are violated, they may feel mistreated. Anticipated stigma, experienced stigma, or traumatizing experiences drive mistrust reluctance to engage in medical systems (37, 38).

A structural vulnerability lens frames the problem of health access inequity around institutions and policies rather than individual choices (15). Solutions for these barriers should focus on awareness (among policy makers) of and efforts to eliminate the structural conditions that produce them rather than placing the burden of change on individuals disproportionally harmed by those structures. By highlighting specific barriers as being especially relevant to inequities, our results suggest priorities for policy-makers wanting to help those most at-risk of harm within our public health infrastructure and healthcare delivery system. Individuals experiencing economic precarity cannot be expected to expend limited resources if doing so risks losing income during isolation. Individuals experiencing unfamiliar or threatening systems of care cannot be expected to engage those systems in order to test. Offering tests through known and trusted healthcare providers improves willingness to test, as do community navigators and peer outreach workers directing people toward available resources. However, policies seeking to ensure widespread participation should also remove threats to precarity by offering free testing in convenient locations, paid sick leave, assistance to reduce the burden of isolating, and privacy protections.

### 4.1 Limitations

Because of difficulty with recruitment, we had limited sampling for some immigrant groups (specifically Latino, Somali, and Arabic). We emphasize that participating community members do not represent the views or experiences of their entire communities. Many community member participants were already engaging with health services when recruited and may have different perspectives than community members not engaging with these services. In addition, most community members from Central Africa were recruited by a single contact associated through a Portland church. Since we have a non-representative sample, we avoid drawing generalized conclusions about testing experiences, perceptions, and factors for any given population group. We focus instead on summarizing the breadth of different testing experiences and influencing factors, and simply noted when a finding came up across all groups or only certain groups.

## 5 Conclusion

The COVID-19 pandemic revealed substantial gaps in our public health infrastructure. To avoid under-testing and its associated public health consequences during the next pandemic, the infrastructure for continued testing access should be developed as soon as possible. Although the RADx-UP program funded free COVID-19 testing during the study period (39), the end of the public health emergency has resulted in poor test access again. To ensure adequate and appropriate testing during a pandemic, rapid tests must be free and available near structurally vulnerable communities and public services. Community members and agencies serving them must be able to find up-to-date and clear information about when and where to test, in multiple languages and appropriate to low health literacy. Working with the Maine Public Health Association we created such a resource in Maine (40). If COVID-infected individuals from underserved populations are required to quarantine, they require protections from loss of income, housing, or services. Public health programs should address the structural conditions we observed to ensure access and mitigate the risks associated with testing.

## Data Availability

If requested, the raw data supporting the conclusions of this article will be made available by the authors, without undue reservation.
