# Supplementary material for: A qualitative study of how structural vulnerability shaped COVID-19 testing behaviors in Portland, Maine
Source: Front Public Health. 2024 Oct 17;12:1433476. doi: 10.3389/fpubh.2024.1433476 (PMC11524922; doi:10.3389/fpubh.2024.1433476)
Supplement: Supplementary file 1 [file Data_Sheet_1.DOCX]

Interview Guide for Participants

Before we start, what do you think this interview is supposed to be about?

[Listen to response, make sure to address any misunderstanding]

I’m going to ask questions about what you think about COVID and things related to COVID like COVID testing and vaccines. I do have some questions about your living situation or about your age, which may feel personal. We can skip questions you don’t want to answer. I won’t ask your name or anything that could be used to figure out who you are because I want to protect your privacy.

This interview can take up to 30 minutes. Do you have that much time right now?

[Listen to response, if participant is rushed to finish, tell them it would be best not to do the interview right now, but we may be around later if they have more time]

To make sure we get everything accurately, this interview will be recorded. Here’s the recorder. It’s not on right now. I will only turn it on when you tell me it’s ok. I will pause it or turn it off if you ask me to.

And again, to be clear, you don’t have to participate in the interview. You can skip a question if you want, and you can stop the interview at any time.

After the interview, I will give you a $20 gift card as a way of saying thank you for taking time out of your day to talk to me.

Do you have any questions?

Alright. I’m going to turn on the recorder now, ok?

1. To start, can you tell me a little about how the COVID-19 pandemic has impacted your life?
2. Have you ever had COVID? Tell me about that.
   1. Is it possible you were infected with the coronavirus at some point without knowing you had it? [Probe to determine thoughts about asymptomatic transmission]
3. Do you think you will end up getting COVID [again] sometime during the next year?
   1. [If not sure] Do you think the chance of you getting COVID is better or worse than 50/50? Why do you say that?
4. Let’s say you started feeling sick. What would you do? [Probe about seeking medical advice/care and testing]
   1. What symptoms would you expect to feel if you had COVID? [Probe for which specific symptoms would suggest COVID vs something else]
   2. If you got COVID, how sick would you imagine getting? Do you think it’s possible that you’d end up having to stay in the hospital? Do you think it’s possible that you could die if you got COVID?
5. Where can you find good information about COVID? (Alternative: Who could you ask to get information you could trust?)
   1. Can you think of places or people that might be giving bad information [about COVID]? What are they?
6. If you thought you might have COVID, can you think of people you’d be worried might catch it from you? [If not, what about people who are older?]Who are you thinking of?
   1. [If not clear] Do you think COVID is dangerous for some people? Who?
7. If you wanted to make sure you didn’t give it to anyone else, what would you do?
   1. Would you be able to isolate from other people? Why/ why not?
   2. Where are you staying?/Do you live with others? Is it actually possible to isolate from other people who live/stay there too?
8. [Only ask if participant cannot easily isolate] There used to be a hotel in Portland where people could stay at for free if they needed to isolate. Have you heard about that? What have you heard/what do you know about it?
   1. [If not familiar] This is a hotel where people who test positive for COVID can stay for 2 weeks in order to isolate. People from the city deliver food so you don’t have to leave, and it doesn’t cost you anything. It was available in the past, but they recently closed it down.
   2. If something like that were available, does that seem like an option for you in case you got COVID? Why/why not? [Probe whether quarantine shelter is *acceptable* option]
9. If you had to isolate from other people, would that hurt your income/ how you make money? Tell me about that.
10. [Only ask if not already answered] If you started feeling sick, and it was symptoms of COVID, would you want to get a COVID test? Why/Why not? [probe to motivation]
11. If you wanted to get tested for COVID, how would you do it? [Probe: what would be involved?]
    1. Have you ever tried to get COVID testing before? What happened?
    2. When you are/were thinking about getting a COVID test, what are/were some of the questions you’ve had?
    3. [If applicable] What would have made it easier for you to get a COVID test?
12. What if you didn’t feel sick at all, but you found out you might have been exposed to COVID? Do you think that would enough of a reason to get tested? Why/why not?
    1. People sometimes talk about “close contact” with COVID. What does that mean to you? What kinds of things would you think about to decide if you had “close contact”? (e.g. how close, how long, masked/not, vaccinated/not, indoor/outdoor)
    2. What if someone at the Maine CDC called you and asked you to get tested because *they* thought you had close contact? Would you get tested in that case? Why/why not?
13. [If applicable] In this interview, you’ve said that you would want to get tested if you thought you might have COVID. Do you think everyone else in your community feels the same way?
    1. [If not] What are some reasons people might be reluctant to get tested?
    2. Some people have said there’s no point in getting tested because doctors can’t give you any medicine for COVID anyway. What do you think about that?
    3. Some people have said they’d rather not get tested because a positive test would mean having to isolate and missing work and friends. What do you think about that?
    4. Some people might not want to get tested because they say the tests aren’t really accurate. Have you ever heard that? What do you think about that?
14. Whenever someone tests positive, it’s supposed to be reported to the CDC. Does that seem like a useful thing to do?
    1. If you were getting tested, how would you feel about giving your information to the CDC?
15. Are you vaccinated? Would you mind telling me about your decision? [probe to motivation]
    1. [As appropriate] What concerns did you have?

Finally, I have some demographic questions we ask everyone. [Add, if needed: Even if it seems obvious, we want to make sure it’s on the transcript.]

1. How old are you?
2. What gender do you identify with?
3. What race do you identify as?
4. Do you identify with any ethnic group? If so which?
5. [For immigrant populations] What country did you emigrate from?
6. Would you like to share anything else about you or your identity that you think is relevant to how you think about COVID and testing? For example, could be your religion or politics or anything really.
7. Those are all my questions. Is there anything else you’d like to say before we end the interview?
