# Supplementary material for: A qualitative study of how structural vulnerability shaped COVID-19 testing behaviors in Portland, Maine
Source: Front Public Health. 2024 Oct 17;12:1433476. doi: 10.3389/fpubh.2024.1433476 (PMC11524922; doi:10.3389/fpubh.2024.1433476)
Supplement: Supplementary file 2 [file Data_Sheet_2.DOCX]

Interview Guide for Key Informants

Thanks for taking the time to talk with me today. The goal of this research is to better understand patient perceptions of, fears about and experiences with COVID-19 testing. We’re focusing on specific populations, including [relevant population for key informant]. We wanted to talk to you because you work closely with this population and are likely to be familiar with their experiences and concerns. We plan to use the information we collect to help ensure that testing programs are sensitive to the concerns of community members.

During the interview, you’ll be asked to share stories you’ve either witnessed or heard about from the people you serve, related to COVID and testing. To be clear, we are not interested in anyone’s name, so please do not say anything that could be used to identify an individual. We will not be identifying any of our participants, including you. If you accidentally share identifying information, we will erase it from the transcript in order to protect confidentiality. Keep in mind that I won’t be asking about your own beliefs about these issues.

The interview is recorded to ensure accuracy. We will not share the recordings beyond the project. If we quote you, you will not be identified.

And finally, this is research and you are under no obligation to participate. You may choose not to answer specific questions or to end the interview any time you choose.

Any questions? If not, with your permission I’ll go ahead and turn on this recorder.

[Turn on the recorder]

| **Table 3: Topic Areas and Sample Questions for the Semi-structured Interview Guide** | |
| --- | --- |
| **Topic** | **Question Areas** |
| **Background** | - To start, tell me about your role. - And today we’ll be discussing what you’ve heard from [population]. How long have you been working with this community? |
| **Beliefs and attitudes around COVID** | - Can you give me a sense of how COVID has impacted this community in the past year? - What are you hearing from clients about their overall concern about getting COVID? Tell me about that. [Probe: range of views, most common] - What are you hearing from clients about their overall concern about spreading COVID to other people? Tell me about that. [Probe: range of views, most common] - What are you hearing from clients about being in crowded places and risk of exposure to COVID? - [For those who work with clients in shelters] What kinds of issues or concerns have you heard from clients in shelters about being exposed to COVID? - [For those who work with clients in other settings] What kinds of issues or concerns have you heard from your clients about being exposed to COVID in their housing? How about at work or other places? |
| Beliefs and attitudes around rapid COVID testing, including bias and stigma | Next I’ll be asking about rapid COVID testing.   - What are the kinds of circumstances where one of your clients would need or want to get tested? - What kinds of experiences have you heard from your clients regarding COVID-19 testing? [Probe: with symptoms vs w/o symptoms] - Are there reasons you have heard that might make a client not want to be tested? What are they? - What do you think doctors or public health officials should know about how people in this community think about testing? - Do you think the clients you work with would have COVID testing if it was offered periodically? Why or why not? - What happens to the clients you work with if they test positive? [Probe to understand full impact] Can you share a story related to this?   - [As applicable] How could situations like this be handled better? |
| Good practices for testing | As I mentioned before, we’d like to understand the best way to implement COVID-19 testing with this community, so I’d like to dig deeper.   - Can you think of any practices around COVID testing that should be changed or avoided, if at all possible, with this community? Why? Can you share a story that speaks to this kind of issue? - Have you heard of any approaches to testing that seem to work better with this group? Tell me about that. - What else could be improved regarding COVID testing in this community? |
| Wrapping up | - Those are most of my questions, but I wonder if there’s anything else you think is relevant to understanding attitudes around testing in this community? - Thinking back over this interview, is there anything you’d like to emphasize or clarify before we end? |
